# Identification of antibiotic resistance genes in *Escherichia coli* from subclinical mastitis milk in dairy cows and goats, East Java Province

**DOI:** 10.17221/80/2023-VETMED

**Published:** 2024-02-28

**Authors:** Desy Cahya Widianingrum, Denada Grace Silaban, Wahyu Indra Duwi Fanata, Himmatul Khasanah

**Affiliations:** ^1^Department of Animal Science, Faculty of Agriculture, University of Jember, Jember, Indonesia; ^2^Department of Agrotechnology, Faculty of Agriculture, University of Jember, Jember, Indonesia

**Keywords:** multiple drug resistant, sulfhydryl variable (*SHV*), temoneira enzyme (*TEM*), zoonosis

## Abstract

Antibiotics are still used to treat mastitis in dairy cows in Indonesia. This study aimed to analyse antibiotic resistance genes in *Escherichia coli* (*E. coli*) from subclinical mastitis milk in East Java Province, Indonesia. The samples consisted of subclinical mastitis milk from cows and goats. A total of 592-quarter cow’s milk and 71 goat’s milk samples from both halves of the udder were collected from 67 farms in Lumajang, Banyuwangi, Malang, Sidoarjo, Jember, Pasuruan, Probolinggo, and Mojokerto. Subclinical mastitis samples were screened using the California mastitis test (CMT). *E. coli* was identified by phenotypic and genotypic methods. *E. coli* was confirmed with a primer specific to the polymerase chain reaction (PCR) technique. Gene resistance of *E. coli* was tested using the multiplex-PCR (mPCR) technique with primers encoding the genes temoneira enzyme (*TEM*), oxacillinase (*OXA*), sulfhydryl variable (*SHV*), and cefotaximase-munich IV (*CTX-M IV*). These genes were chosen because mastitis treatment generally uses oxacilline and β-lactam antibiotics. All data obtained were analysed descriptively. The results show that six isolates of *E. coli* (46.15%) carried a single resistance gene (*TEM* or *SHV*) and two isolates (33.33%) were confirmed as multiple drug-resistant organisms (MDROs) (*TEM* and *SHV*). The resistance genes were found in samples originating from Blitar, Banyuwangi, Lumajang, and Pasuruan Regencies. This research implies that antibiotic-resistance genes found in *E. coli* on certain farms are dangerous and may allow gene transmission to other bacteria that make treatment for mastitis or other bacterial infections ineffective.

East Java is a province with the highest dairy cow population in Indonesia. Based on data from Statistics Indonesia (2022), it is known that in East Java, the population of dairy cows is 314 385 from a total national 592 897 heads, and the population of goats is 3 897 185 from a total national 19 397 960 heads. This number increased by approximately 2% from the previous year. Regrettably, the total milk production from dairy livestock in Indonesia was insufficient for national needs. Milk production in East Java was 543 687.16 tons and national production was 968 980.14 tons (Statistics Indonesia 2022). Based on the needs of the human population in Indonesia, this number is still very low. The average per capita milk consumption per year is 16.9 g.

One of the factors causing low milk production is the presence of disease (Biobaku and Amid 2018), especially mastitis. There are two forms of mastitis based on the physical symptoms. Clinical mastitis is udder inflammation accompanied by obvious physical symptoms, whereas subclinical mastitis is not (Kalinska et al. 2017). The prevalence of subclinical mastitis in East Java Province is very high, reaching 67% (Widianingrum et al. 2022).

Pathogens causing mastitis include bacteria, viruses, moulds, and algae from the environment and they spread also via livestock-to-livestock transfer (Ali et al. 2022). They include e.g. Gram-positive bacteria such as *Staphylococcus aureus (S.* *aureus), Staphylococcus epidermidis,* and other non-aureus *Staphylococci* (Widianingrum et al. 2022) and Gram-negative bacteria such as *Escherichia coli* (*E. coli*), *Proteus mirabilis*, *Klebsiella* spp., and *Citrobacter* spp. (Sierra et al. 2023).

Treating mastitis caused by *E. coli* is often difficult because it can produce extended-spectrum beta-lactamases (ESBL) enzymes (Prasetya et al. 2019). ESBL is a class of beta-lactamase enzymes located in plasmids or chromosomes that control genes producing lactamase (Masruroh and Sudarwanto 2016). These enzymes are the Temoneira enzyme (*TEM*), oxacillinase (*OXA*), sulfhydryl variable (*SHV*), and cefotaximase-munich (*CTX-M*) (Lai et al. 2022). Sierra et al. (2023) reported that due to these enzymes, *E. coli* is resistant to antibiotics. In Indonesia, dairy farmers still use antibiotics, so we hypothesised that resistant bacteria spread in various regions. In this study, we performed the resistance gene mapping in cases of subclinical mastitis caused by *E. coli* infection in East Java Province.

## MATERIAL AND METHODS

The milk samples were obtained from lactating dairy cows, and each farmer carried out the milking according to their operational procedures.

### Sampling of subclinical mastitis milk

The sample collection consisted of subclinical mastitis milk from cows and goats in East Java Province.

A total of 592 quarter cow’s milk samples and 71 goat’s milk samples from both halves of the udder were collected from 67 farms in Lumajang, Banyuwangi, Malang, Sidoarjo, Jember, Pasuruan, Probolinggo, and Mojokerto. Subclinical mastitis samples were screened using the California mastitis test (CMT).

### Isolation and identification of *Escherichia coli*

Positive CMT samples were grown on selective media Eosin Methylene Blue Agar (EMBA) (Oxoid, Hampshire, United Kingdom) and tested for their biochemical properties. The non-resistance bacteria as a negative control in this study was *E. coli* BL21 from the Agrotechnology Laboratory, Faculty of Agriculture, University of Jember, Indonesia. Colony characters referring to *E. coli* were isolated from DNA using the DNA Extraction Kit (Geneaid Biotech, New Taipei City, Taiwan). A molecular-based determination for particular *E. coli* isolates was done using polymerase chain reaction (PCR) (Bio-Rad Laboratories, Hercules, CA, USA). Briefly, the DNA of *E. coli* was isolated following Windria et al. (2016) and subjected to a standard cycle PCR condition using *E. coli* species-specific primers with *16S rRNA* primers (Table 1).

**Table 1:** Oligonucleotide primers coding *E. coli* and antibiotic-resistance genes

Gene	Primer sequence	PCR programme	Target size	Reference
*16S rRNA*	GGG AGT AAA GTT AAT ACC TTT GCT C TTC CCG AAG GA CAT TCT	1	584 bp	Radji et al. (2010)
*TEM*	AGT GCT GCC ATA ACC ATG AGT G CTG ACT CCC CGT CGT GTA GAT A	2	431 bp	Kim et al. (2009)
*OXA*	ATT ATC TAC AGC AGC GCC AGT G TGC ATC CAC GTC TTT GGT G	2	296 bp	
*SHV*	GAT GAA CGC TTT CCC ATG ATG CGC TGT TAT CGC TCA TGG TAA (integrated DNA technologies); *CTX-M IV* forward primer	2	214 bp	
*CTX-M IV*	GAC AAA GAG AGT GCA ACG GAT G TCA GTG CGA TCC AGA CGA AA	2	501 bp	

Programme 1 = pre-denaturation: 95 °C for 5 min, denaturation: 94 °C for 20 s (35 cycles), annealing: 56 °C for 20 s, elongation: 72 °C for 30 s, final elongation: 72 °C for 10 min; Programme 2 = pre-denaturation: 94 °C for 5 min, denaturation: 94 °C for 1 min (30 cycles), annealing: 61 °C for 1 min, elongation: 72 °C for 1 min, final elongation: 72 °C for 5 minutes

PCR mixture consisting of 10 μl PCR Master Mix 2X My *Taq* HS Red Mix (Bioline, London, UK), two μl primer reverse, two μl primer forward, four μl nuclease-free water, and two μl genomic DNA templates. PCR results were analysed on 2% agarose (Promega, Madison, USA) with FloroSafe DNA Stain two μl (Axil Scientific Pte Ltd, SCoence Park Road, Singapore). Four μl of PCR results and three μl of 100 bp ladder (Thermo Scientific, Lietuva, Lithuania) were placed into the gel wells and then electrophoresed with an electric current of 100 volts for 30 minutes. Electrophoretic DNA bands were observed using a UV transilluminator (Major Science Co., Ltd., Taoyuan City, Taiwan).

### Determination of antibiotic-resistance genes in *Escherichia coli*

Identification of resistance genes in *E. coli* was carried out using multiplex-PCR (mPCR) technique with primers temoneira enzyme (*TEM*), oxacillinase (*OXA*), sulfhydryl variable (*SHV*), and cefotaximase-munich IV (*CTX-M IV*) (Table 1). The following presents the composition of mPCR reagents for detecting antibiotic-resistance genes (Table 2).

**Table 2:** Composition of multiplex PCR reagents for detection of antibiotic resistance genes

Reagent		Volume (μl)
Mastermix PCR		10 μl
Primer		
*CTX-M IV*	forward	1 μl
*CTX-M IV*	reverse	1 μl
*TEM*	forward	1 μl
*TEM*	reverse	1 μl
*OXA*	forward	1 μl
*OXA*	reverse	1 μl
*SHV*	forward	1 μl
*SHV*	reverse	1 μl
DNA template		2 μl
Total volume		20 μl

### Antibiotic resistance gene mapping

For antibiotic resistance gene mapping, ArcGIS v10.8 (Environment Science & Research Institue, India) was used.

### Data analysis

The data obtained were analysed and presented qualitatively using percentages, tables, and figures.

## RESULTS AND DISCUSSION

Subclinical mastitis is diagnosed by the California mastitis test (CMT). Alkyl aryl sulfonate (a component of CMT) is highly sensitive to the number of nuclei of somatic cells or leukocytes. Somatic cells will react with CMT reagents, marked by the appearance of a gel (Sevitasari et al. 2019). The present study shows that subclinical mastitis cases in East Java are relatively high, reaching more than 60% in both cattle and goat milk samples. There were 397 from 592 (67.06%) cow’s milk samples and 47 from 71 (66.19%) goat’s milk positive for CMT.

**Figure 1:**
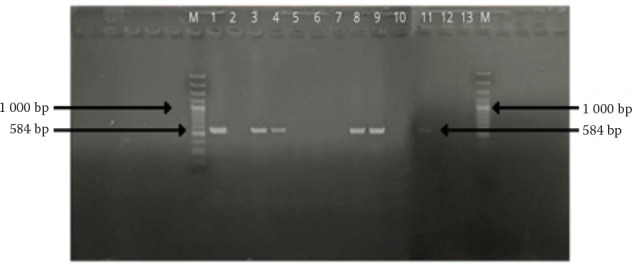
Results of electrophoresis of DNA on the *16S rRNA* gene (*E. coli*) with an amplicon size of 584 bp using agarose gel Ket: M = marker/ladder DNA 100 bp; Lanes 1–13 = sample, E 24 (lane 1), E 17 (column 3), E 36 (lane 4), EK 27 (lane 8), KE 34 (lane 9), E 34 (lane 11)

Based on the microbiological examination, a total of 13 (2.9%) (suspected) *E. coli* bacteria were found in cow and goat milk samples. To validate the bacteria, we confirmed it with the genotypic methods and we identified six *E. coli* strains in this study (Table 3, Figure 1). The *E. coli* was distributed in four areas of East Java province. Determination of the bacteria that cause mastitis is done by microbiological test, and confirmation can be done using PCR assay (Widianingrum et al. 2022).

**Table 3:** Distribution percentage of *E. coli* isolated from cow’s and goat’s milk in East Java

Region	Number of isolates	Phenotypic proportion (%)		Positive number *16S rRNA*	Genotypic proportion*16S rRNA* (%)	
		cow	goat		cow	goat
Lumajang	3	2 (4.26%)	1 (1.4%)	1	1 (7.69%)	–
Banyuwangi	2	2 (4.26%)	–	1	1 (7.69%)	–
Pasuruan	4	4 (8.51%)	–	2	2 (15.38%)	–
Sidoarjo	1	1 (2.13%)	–	–	–	–
Blitar	3	–	3 (4.17%)	2	–	2 (15.38%)
Total	(13/444)	(9/13)	(4/13)	(6/13)	(4/9)	(2/4)
Percentage	2.9%	69.23%	30.77%	46.15%	44.44%	50%

There was no interaction between the CMT score and the presence of *E. coli* bacteria in subclinical mastitis cases in this study (Table 4). This is an important finding in our research because, at a low score (CMT + 1), *E. coli* bacteria were found which can harm livestock and milk consumers. *E. coli* bacteria in subclinical mastitis milk in this study were found in Lumajang, Banyuwangi, Sidoarjo, and Blitar samples. This fact can be useful for farmers to treat their livestock diagnosed with mastitis immediately even though the CMT score is low (CMT + 1).

**Table 4:** CMT results, phenotypic, and genotypic identification of *E. coli*

Regency	Positive CMT score	Positive phenotypic	Positive genotypic
Lumajang	+++	E 17	✓
	+++	E 18	–
	+	EK 8	–
Banyuwangi	++	E 23	–
	++	E 24	✓
Pasuruan	+++	E 34	✓
	+++	E 35	–
	+++	E 36	✓
	+++	E 37	–
Sidoarjo	+++	E 40	–
Blitar	++	EK 27	✓
	+	EK 33	–
	+	EK 34	✓

E = cow’s milk sample; EK = goat’s milk sample

We used *16S rRNA* in this study because this gene is most frequently applied as a molecular marker compared to the other two types of ribosomal RNA, namely *5S* *rRNA* and *23S* *rRNA*. Generally, *16S rRNA* is more stable and has a hypervariable region which is a particular area for identifying genus and species (Noer 2021).

**Figure 2:**
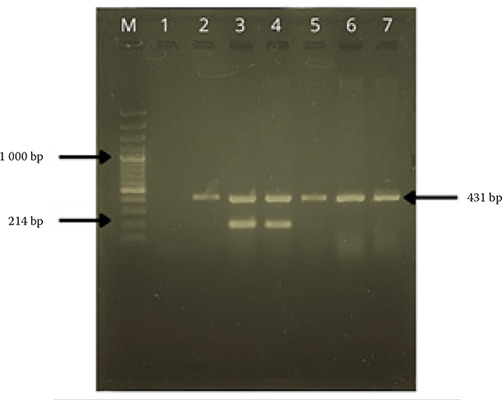
DNA electrophoresis results for antibiotic resistance genes (*TEM*, *SHV*, *CTX-M IV*, *OXA*) Marker/ladder DNA 100 bp; Column 1 = negative control of *E. coli* BL21; Column 2,5,6,7 = *TEM* positive cow’s milk sample; Column 3,4 = *TEM* and *SHV* positive goat’s milk sample

Based on the distribution of antibiotic resistance gene data (Table 5, Figure 2), we found four (66.67%) isolates of bovine *E. coli* expressing *TEM* genes, and there were no other genes. In goat’s milk, there were two isolates (33.33%) of *E. coli* that had the *TEM* gene, two isolates (33.33%) having the *SHV* gene, and two isolates (33.33%) having the *TEM* + *SHV* genes. These findings indicate that the *E. coli* bacteria that cause mastitis in our studied area have resistance traits that encode the *TEM* or *SHV* gene or multiple genes (*TEM* and *SHV*). Based on the international expert proposal for Interim Standard Definitions for Acquired Resistance, if there is an incidence of resistant isolates to at least one antibiotic from more than three groups of antibiotics, then multiple drug resistance organisms (MDRO) have occurred (Basak et al. 2016). Several pathogens, including MDROs, include methicillin-resistant *S. aureus* (MRSA), vancomycin resistance enterococcus (VRE), certain Gram-negative bacilli (GNB) including *Enterobacteriaceae* with plasmid encoded extended-spectrum beta-lactamases (ESBL). In the last decade, MDROs have shifted from Gram-positive to Gram-negative bacteria. This is due to the emergence of Gram-negative bacteria resistant to various antibiotics (Bhattacharya 2013).

**Table 5:** Distribution of antibiotic resistance genes in cow’s and goat’s milk samples

Isolate code	Source	*16S rRNA*	Antibiotic resistance genes				
			*TEM* (%)	*SHV* (%)	*TEM* and *SHV* (%)	*CTX-M IV* (%)	*OXA* (%)
E 24	cow	(+)	(+)	–	–	–	–
E 17		(+)	(+)	–	–	–	–
E 36		(+)	(+)	–	–	–	–
E 34		(+)	(+)	–	–	–	–
Total		(4/13) 30.77%	(4/6) 66.67%	0	0	0	0
EK 27	goat	(+)	(+)	(+)	(+)	–	–
EK 34		(+)	(+)	(+)	(+)	–	–
Total		(2/13) 15.38%	(2/6) 33.33%	(2/6) 33.33%	(2/6) 33.33%	0	0

**Figure 3:**
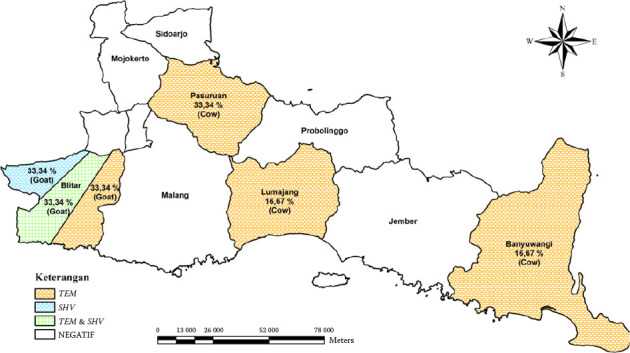
Antibiotic resistance gene mapping of *E. coli* from cow’s and goat’s milk originating from East Java *OXA* = oxacillinase; *SHV* = sulfhydryl variable; *TEM* = temoneira

The distribution of resistance genes possessed by *E. coli* in this study is depicted in Figure 3.

Four samples carrying the *TEM* gene were found in the specimens isolated from Banyuwangi, Lumajang, and two others from Pasuruan. The inappropriate use of antibiotics in treating infected animals can increase the case numbers of antibiotic resistance (Kraemer et al. 2019). Low concentrations of antibiotics (subtherapeutic) can encourage genetic modifications that develop resistance (Ventola 2015).

The prevention step is to start mitigating MDRO infections. Farmers should be more careful in using antibiotics (e.g. efficiency of indication, dosage, duration, and use of antibiotics as medicine) (Ministry of Health of the Republic of Indonesia 2015).

However, using antibiotics for the treatment of mastitis also requires attention because of their residues in milk and meat. Usually, the minimum withdrawal time for antibiotics is five days after therapy, or 13 days for broad-spectrum antibiotics (Meutia et al. 2016).

In terms of numbers, our findings are in the minority. But, resistance genes can be transmitted, causing the spread of resistance traits (Batabyal et al. 2018). The environment acts as the origin of resistance. This is because only 10–80% of antibiotics are metabolised, and the rest is excreted as active compounds through urine and faeces into the environment, which can make environmental microbes resistant (Food and Agriculture Organization 2018). Humans, animals, and the environment are interconnected, and bacterial transfer, including mobile genetic elements (MGEs) (plasmids and transposons) between species is easy (Woolhouse et al. 2015).

This study implies that dairy products from several farms can spread antibiotic-resistant pathogens so that they have the potential to transmit zoonoses (Khusnan et al. 2016). Therefore, information on the prevalence of resistance gene distribution is precious in understanding the distribution mechanism in several regions and as an evaluation of the mastitis treatment process carried out by breeders (Han et al. 2022). Infectious diseases caused by resistant bacteria result in prolonged illness, increasing the risk of death, failed treatment or livestock becoming a carrier (Humaida 2014).

In conclusion, it can be stated that resistance genes have been found in *E. coli* from milk infected with subclinical mastitis in goats and cattle in Lumajang, Banyuwangi, Sidoarjo, and MDRO in Blitar, East Java. The information provided in this research is critical to identifying gaps in knowledge that will guide future studies to develop therapeutic interventions for mastitis.
